# Exploring the impact of smartphone addiction on decision-making behavior in college students: an fNIRS study based on the Iowa Gambling Task

**DOI:** 10.3389/fpsyt.2024.1342521

**Published:** 2024-04-22

**Authors:** Xiaolong Liu, Ruoyi Tian, Xue Bai, Huafang Liu, Tongshu Li, Xinqi Zhou, Yi Lei

**Affiliations:** Institute of Brain and Psychological Sciences, Sichuan Normal University, Chengdu, China

**Keywords:** smartphone addiction, decision making, college student, IGT, fNIRS

## Abstract

The pervasive use of smartphones, while enhancing accessibility to information and communication, has raised concerns about its potential negative effects on physical and mental health, including the impairment of decision-making abilities. This study investigates the influence of smartphone addiction on decision-making in college students. A sample of 80 individuals aged 17 to 26 was selected and divided into two groups based on their Smartphone Addiction Scale-Short Version (SAS-SV) scores. Participants underwent the Iowa Gambling Task (IGT) to evaluate their decision-making in risky and uncertain conditions, while fNIRS recorded their prefrontal cortex activity. The study found that individuals prone to smartphone addiction tend to make riskier choices in risky situations. However, when faced with decisions based on ambiguity, the smartphone addiction group showed increased brain activity in the dlPFC (specifically in channels 4, 9, and 11) compared to when making risky decisions. Despite this increased brain activation, there was no observable difference in behavior between the addiction-prone and control groups in ambiguous scenarios. Notably, the left dlPFC (e.g., channel 4) exhibited significantly higher activation in the addiction group compared to the control group. Findings suggest that smartphone addiction can detrimentally influence decision-making, behaviorally and neurologically, particularly in uncertain contexts. This study supports the classification of smartphone addiction as a genuine addiction and underscores its significance in psychiatric research. In essence, our research underscores the adverse effects of excessive smartphone use on decision-making processes, reinforcing the necessity to treat smartphone addiction as a pressing public health issue.

## Introduction

Smartphone Addiction (SA) has emerged as a significant psychosocial issue in the digital era, characterized by excessive and compulsive smartphone use. This phenomenon is garnering attention due to its detrimental impact on individuals’ well-being (1, 2). Those affected by SA often suffer from diminished endurance and focus during smartphone use, experience withdrawal symptoms such as distress when separated from their devices, neglect other life activities, and feel a loss of control over their usage despite being aware of its negative consequences (3–5).

The ubiquity of smartphones as the primary means for internet access has led to an escalation in their overuse, with some users developing an unhealthy dependency (3, 6). According to the China Internet Network Information Center (CNNIC), by December 2021, the number of mobile internet users in China had surpassed a billion, nearly all of whom accessed the internet via smartphones. College students, in particular, have been identified as a demographic at high risk for SA. For instance, a study among Chinese medical students found an alarming SA prevalence rate of 52.8% (7). Other studies corroborate the widespread nature of problematic smartphone use among this group (8–10). The implications of SA are far-reaching, contributing to mental health issues such as depression, sleep disorders, social anxiety, and notably, compromised decision-making abilities (11–15). Decision-making is a critical element in understanding addictions (16). including SA, which is often classified as a behavioral addiction (3, 5). Research has consistently shown that decision-making is adversely affected in various forms of behavioral addiction. Therefore, investigating decision-making functions is essential in exploring the full spectrum of SA’s impact (17–19).

Decision-making is an intricate cognitive process that necessitates choosing among various actions by evaluating the expected value or utility of their potential outcomes (20, 21). This process significantly influences the direction of an individual’s life path. However, decision-making is often clouded by uncertainty. In such contexts, we can identify two distinct forms of decision-making: under risk and under ambiguity. Decision-making under risk is characterized by situations where the precise probabilities of outcomes are known (22). while decision-making under ambiguity pertains to scenarios where these probabilities are indeterminate (23). In the complexities of daily life, individuals are likely to encounter both risk and ambiguity, necessitating judicious choices in light of the circumstances at hand.

To investigate decision-making, researchers have developed a variety of methodologies. Among the commonly employed behavioral experimental paradigms are the Wheels of Fortune (24), the Balloon Analogue Risk Task (BART) (25), and notably, the Iowa Gambling Task (IGT) (26). The IGT is a prominent laboratory tool designed to evaluate decision-making capabilities, capturing the essence of real-life choices by integrating rewards and punishments under uncertain conditions. Further studies have illustrated that the IGT effectively encompasses scenarios involving both ambiguous and risky decision-making (27), rendering it a valuable instrument for probing the intricacies of decision-making processes.

Decision-making is a multifaceted cognitive process that engages several brain regions, notably the prefrontal cortex (PFC) (28). Research utilizing functional near-infrared spectroscopy (fNIRS) has shown that the dorsolateral prefrontal cortex (dlPFC) reflects subjective value and is linked to risk attitudes (29), while functional MRI (fMRI) studies indicate a decrease in ventromedial prefrontal cortex (vmPFC) activity with increased risk (30). Additionally, event-related potential (ERP) studies have associated the feedback-related negativity (FRN) with value evaluation in risk decision-making, noting a heightened sensitivity to negative feedback (31).

The decision-making abilities of individuals with smartphone addiction in ambiguous situations are notably weaker than those of healthy controls, yet in risky situations, differences are less apparent (14). Contrarily, some research suggests that smartphone addiction correlates with impaired decision-making, marked by poor impulse control (32, 33). These individuals often favor immediate rewards over long-term outcomes, potentially perpetuating addictive behaviors. Neuroimaging studies have provided additional insights, revealing that smartphone addicts show diminished skin conductance responses when facing losses, yet heightened responses upon receiving rewards (14). Moreover, neuroimaging has demonstrated greater activation in the right medial prefrontal cortex (mPFC) during advantageous choices in ambiguous situations, with similar activation levels for all choices under risk (23). In gamblers, the dlPFC shows increased responsiveness to high-risk disadvantageous choices (28), underscoring distinct neural activation patterns in individuals with behavioral addictions compared to their healthy counterparts across different decision-making scenarios.

fNIRS is a non-invasive method that tracks cortical blood flow and oxygenation changes, offering valuable insights into brain activity (34). Its portability, ease of use, and balanced spatial and temporal resolution make it particularly useful for monitoring neural responses during decision-making tasks such as the Iowa Gambling Task (IGT) (35). While technological advancements have made information more accessible, excessive smartphone use is associated with a host of health issues (36). Smartphone addiction (SA), although not yet uniformly defined, is recognized as a behavioral addiction with symptomatology similar to that of compulsive gambling and substance abuse as outlined in DSM-5 (37). Given the significant harm SA can cause, it is crucial to understand its effects, particularly on decision-making in college students.

This study examines the behavioral and physiological impacts of SA on college students’ decision-making. We propose that those with SA will display more impulsive behavior and weaker decision-making in ambiguous situations, but no discernible difference in risky scenarios compared to their non-addicted peers.

Physiologically, we expect to see divergent neural responses during ambiguous decision-making tasks, with the addiction group showing increased brain activation for advantageous choices. In contrast, we predict similar brain activation patterns between groups in risky decision-making.

To explore these hypotheses, we enlisted 80 college students aged 18 to 25 to participate in the IGT while undergoing fNIRS scanning. Our research aims to shed light on the behavioral and neurological effects of smartphone addiction on decision-making among college students.

## Methods

### Participants

Our study enlisted 80 college students from a university campus, balanced in gender with 39 females (average age 21 ± 1.9 years) and 41 males (average age 19 ± 1.7 years). All were right-handed, with normal or corrected vision, normal hearing, and no history of brain injury, substance addiction, or mental disorders.

Before the experimental tasks, participants completed a series of psychological assessments. The Smartphone Addiction Scale - Short Version (SAS-SV) (3) gauged levels of smartphone addiction. Emotional states were measured using the Positive Affect and Negative Affect Scale - revised version (PANAS), while impulsiveness was evaluated with the Barratt Impulsiveness Scale-11 (BIS-11) (38). The Beck Depression Inventory-II (BDI-II) (39) and the Beck Anxiety Inventory (BAI) (40) assessed depressive symptoms and anxiety levels, respectively. These instruments helped profile the psychological background of the participants.

Ethical clearance for the study was granted by the Institutional Review Board of Sichuan Normal University. Informed consent was obtained in writing from all participants before they engaged in the study activities.

### Behavioral methods

#### The Iowa Gambling Task

**Figure 1:**
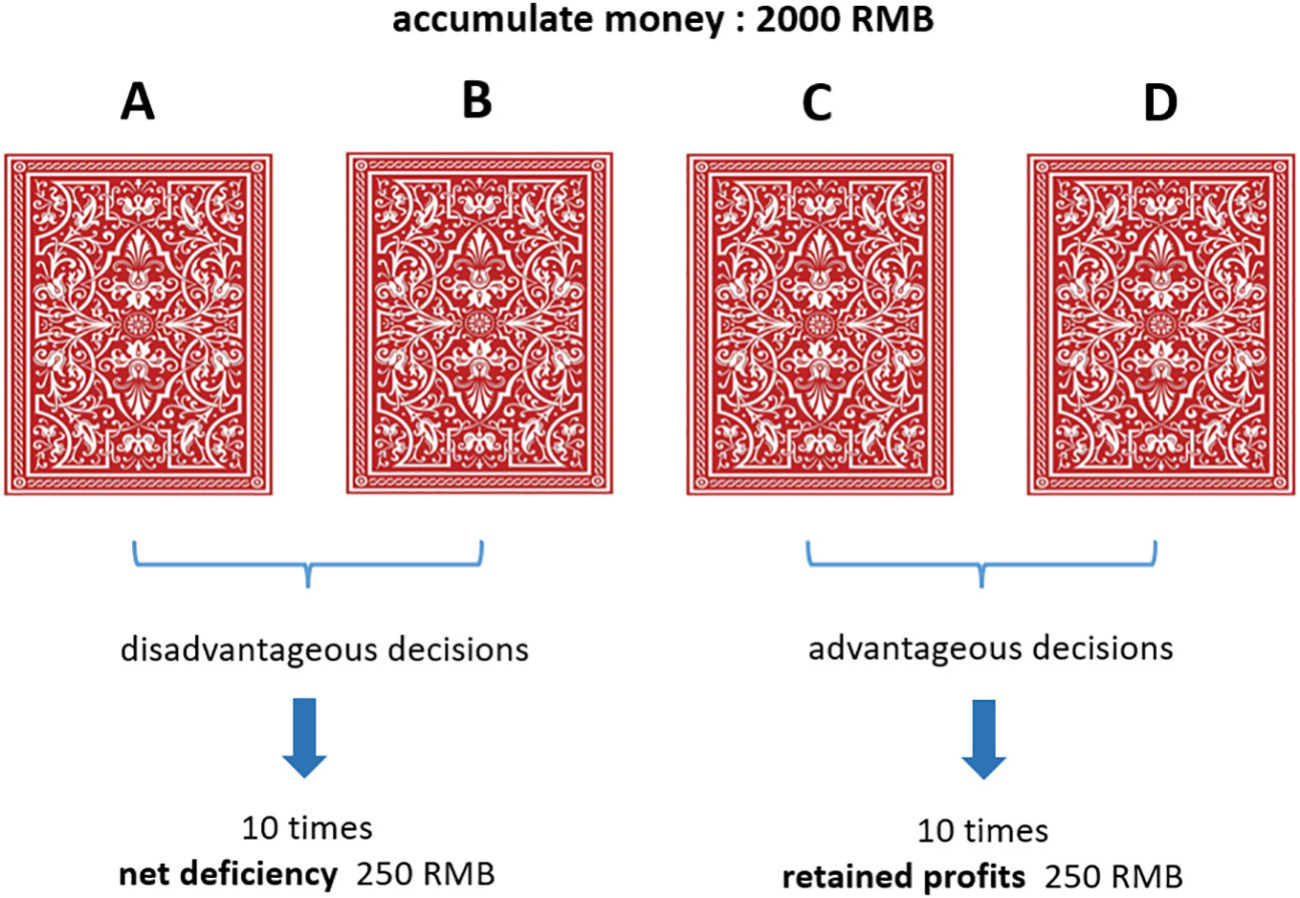
Schematic diagram of IGT experimental paradigm task.

In our study, we utilized the Iowa Gambling Task (IGT) (26) to examine decision-making processes. The task involved four virtual cards labeled A, B, C, and D (**Figure 1**), displayed on a computer screen. Participants started with a loan of 2000 yuan and were tasked to maximize profits by selecting cards over 100 trials, with their final reward tied to their performance.

Each card choice resulted in variable monetary gains and losses, simulating real-life financial decisions. Cards A and B were “disadvantageous,” offering high rewards but steeper penalties, while cards C and D were “advantageous,” with smaller rewards but also reduced losses. Unbeknownst to the participants at the start, choosing disadvantageous cards typically led to a net loss, whereas opting for advantageous cards resulted in net gains. Research has shown that participants can learn and adapt to the rules of the Iowa Gambling Task (IGT) over time (41). The IGT is characterized by two distinct learning phases: the initial phase, which covers roughly the first 40 trials, involves decision-making based on implicit learning, as participants are not yet aware of the specific contingencies guiding their choices. In the subsequent phase, which begins after the initial trials and varies among individuals, participants develop a conceptual understanding of the task, and their choices become more influenced by explicit knowledge of the risks tied to each card deck. This advanced stage is closely linked to higher executive functions such as categorization, monitoring, and cognitive flexibility. As such, the early IGT trials represent decision-making under ambiguity, while the later trials involve decision-making under risk (27).

#### fNIRS instrumentation and data acquisition

**Figure 2:**
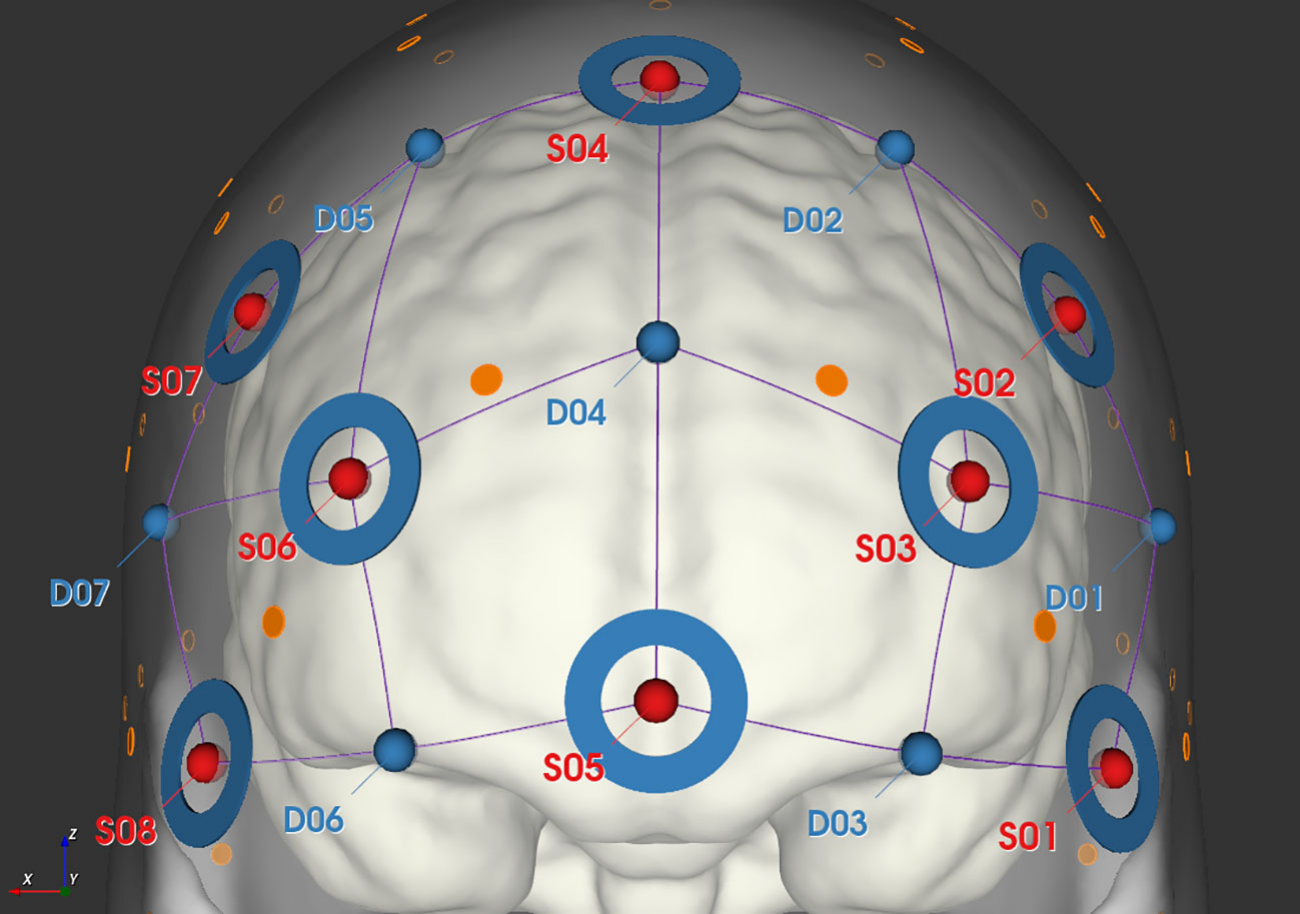
Schematic diagram of fNIRS probe layout, where red dots represent the locations of light sources, blue dots represent the locations of detectors, connecting lines represent the channels, and blue circles indicate the placement of short channels.

We monitored changes in the prefrontal cortex (PFC) oxygenated (HbO) and deoxygenated (HbR) hemoglobin concentrations using the NIRSPORT 2 continuous-wave fNIRS system (NIRx Medizintechnik, Berlin, Germany). A headcap with 8 emitters and 7 detectors, separated by 3 cm, was placed on the participant’s head. These emitters produced light at 760 nm and 850 nm wavelengths, with the data captured at 10 Hz. The optodes were positioned according to the international 10-20 system, targeting the PFC as depicted in **Figure 2**. To control for blood flow artifacts in the scalp and skull, we used short-separation reference channels set 8 mm apart.

#### Procedure

The study was conducted in two main phases. Initially, participants were classified into a smartphone addiction group or a healthy control group based on their scores on the SAS-SV. Upon arrival in the lab, they provided informed consent and completed a series of questionnaires, including demographic details, the PANAS, the BIS-11, the BDI-II, and the BAI for psychological profiling.

**Figure 3:**
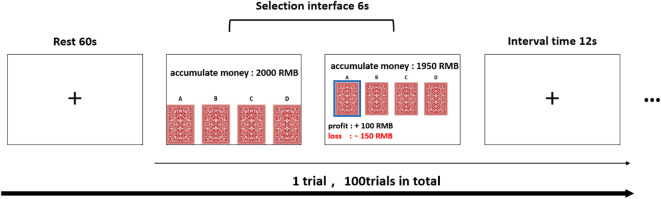
Schematic diagram of IGT experimental paradigm.

The experimental phase involved near-infrared spectroscopy data collection from the orbitofrontal cortex (OFC) and dorsolateral prefrontal cortex (dlPFC) during five blocks of the task, each containing 20 trials. Participants had a 60-second rest before each block. The computer interface displayed ongoing totals and the outcomes of their choices in the upper section, and four card backs in the lower section. Participants used the mouse to make selections within a 6-second window, with the computer making a random choice if they failed to do so in time. A 12-second pause was set between choices, and the experiment lasted about an hour. The procedural flow is illustrated in **Figure 3**.

### Data analysis

#### Behavioral data

To assess differences in psychological profiles between the smartphone addiction and healthy control groups, we conducted independent *t*-tests with the SAS-SV scores defining group membership, and scores from other questionnaires serving as dependent variables. Consistent with the methodology of (42), the IGT performance was quantified for each participant by calculating the net score of advantageous minus disadvantageous choices [(C + D) - (A + B)] per block. These net scores were averaged across participants for each block. We then subjected the IGT net scores from all five blocks (within-subject factor) to a repeated measures analysis of variance (*rmANOVA*) to investigate score patterns over time.

#### fNIRS data

Data processing was conducted using HOMER3, an open-source package compatible with Matlab R2017b. The initial steps of preprocessing involved transforming light intensity data into optical density metrics. This was followed by band-pass filtering within the range of 0.01–0.5 Hz to remove extraneous physiological noise. The conversion of optical density data to hemoglobin concentration changes was achieved through the application of the modified Beer-Lambert law, adopting a path length factor as recommended by prior study for both HbO and HbR (43). To mitigate motion artifacts, spline interpolation was employed within HOMER3 after the conversion process. In the analysis phase, to ensure the exclusion of non-neuronal signals, only data from short channels were considered in the principal component analysis.

The HRF is indeed typically convolved with a design matrix that outlines the experimental conditions. This convolution is then integrated into the General Linear Model (GLM) framework within HOMER3 software, where we employ ordinary least squares to estimate the *beta* values. These values are crucial for subsequent statistical analyses and for generating activation maps using EasyTopo software (44).

Additionally, we gathered data on two categories, HbO and HbR, which reflect hemodynamic changes occurring during the task, as measured by the NIRS channels. In line with prior studies (45, 46), our analysis primarily concentrated on variations in HbO levels. This focus is grounded in the established understanding that HbO serves as the most sensitive marker for detecting alterations in regional cerebral blood flow (rCBF) within the context of NIRS measurements, as highlighted by prior study (47). Therefore, the activation maps of brain regions drawn based on HbO data.

In analyzing the fNIRS data, we employed a 2 × 2 × 2 repeated measures ANOVA (rmANOVA) framework, which dissected the data across two decision-making scenarios (ambiguous *vs*. risky), two types of choices (advantageous *vs*. disadvantageous), and two groups (smartphone addiction (SA) group *vs*. healthy control group). Beta values served as our dependent variables, with decision-making scenarios and choices acting as within-subject factors, and group designation as the between-subject factor. Upon detecting significant interactions, we proceeded with simple effects analysis for in-depth *post hoc* explorations. To uphold the integrity of our statistical analysis, especially when dealing with multiple comparisons that spanned both fNIRS channel-based and behavioral data, we applied the Bonferroni correction method. This adjustment was crucial to ensure that the significance levels of our tests remained stringent and reliable.

### Statistical analyses

This study employed a variety of statistical analyses to examine the data collected. Among these, Pearson correlation analysis was utilized to explore the relationships between different variables of interest, such as the extent of smartphone usage and specific cognitive or behavioral outcomes. Furthermore, we conducted repeated measures Analysis of Variance (rmANOVA) to investigate within-subject effects over conditions and between-group differences. This was complemented by independent samples *t*-tests, as appropriate, for comparing demographic and other categorical variables between the smartphone addiction group and the control group. All statistical tests were performed using SPSS21 sofware, with a significance level set at (*p* < 0.05). Assumptions for each test, including normality and homogeneity of variances, were checked and met unless otherwise stated. Where necessary, data transformations or non-parametric tests were employed. Details on the specific application of these analyses, including the variables involved and the rationale for their use, are provided to ensure clarity and facilitate replication of our study by other researchers.

## Results

### Questionnaire results

A comparison of scores from various psychological questionnaires between two groups of participants was conducted using SAS-SV as the grouping variable. An independent student *t*-test was performed with the other questionnaire indicators as the test variables. The results revealed significant differences between the SA group and the healthy control group in smartphone addiction (*t* = 12.035, *p* <.001), impulsivity (*t* = 2.284, *p* <.05), attentional impulsivity sub-dimension (*t* = 3.891, *p* <.001), and depression (*t* = 3.347, *p* <.001), as presented in **Table 1**. Moreover, there were no significant differences in scores of other variables between the SA group and the healthy control group.

**Table 1:** Demographic information and scores on relevant scales.

	AG(N=41)	HC(N=39)	*t*	*p*
Gender	1.51 ± 0.506	1.51 ± 0.506	-0.006	0.996
sas-sv	42.66 ± 7.296	26.67 ± 4.269	12.035	<0.001
PA	25.49 ± 5.259	26.87 ± 5.307	-1.171	0.245
NA	18.98 ± 6.425	16.85 ± 5.779	1.559	0.124
BIS	59.80 ± 8.588	56.13 ± 5.559	2.284	0.025
Attentional Impulsivity	14.83 ± 2.459	12.82 ± 2.138	3.891	<0.001
Motor Impulsivity	21.39 ± 3.255	20.15 ± 3.192	1.714	0.09
Non-planning Impulsivity	23.59 ± 4.690	23.15 ± 3.083	0.489	0.627
BDI	10.93 ± 7.702	5.74 ± 5.994	3.347	0.001
BAI	33.73 ± 9.309	30.85 ± 6.923	1.567	0.121

Pearson correlation analysis was conducted to examine the relationship between the scores of each psychological scale and smartphone addiction. The results revealed a marginally significant positive correlation between cell phone addiction and negative emotions (*r* = .213, *p* = .058). There was also a significant positive correlation between cell phone addiction and impulsivity as well as its three dimensions (rBIS = .466, *p* <.001). Additionally, a significant positive correlation was found between cell phone addiction and depression (*r* = .482, *p* <.001). However, no significant correlations were observed between smartphone addiction and positive emotion, negative emotion, and anxiety.

### Behavioral results

**Figure 4:**
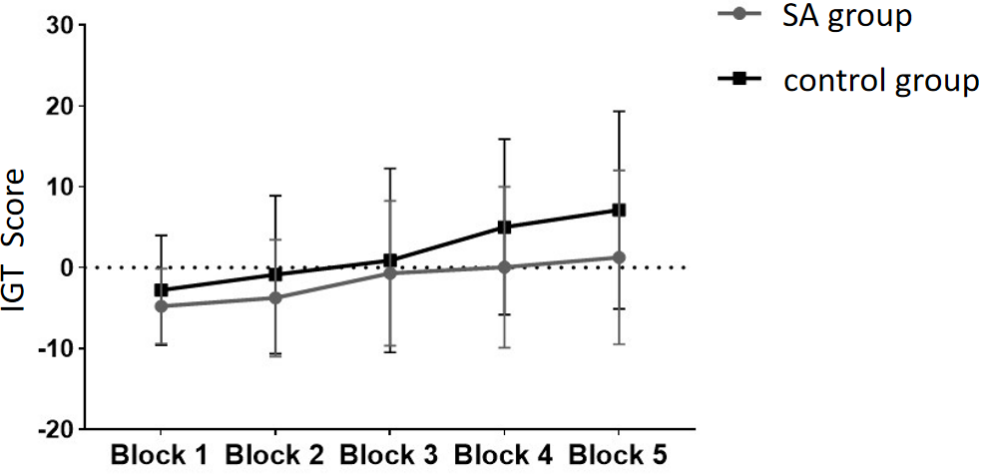
The scores of the SA group and the control group at different stages of the IGT experiment.

Following the integration and unification of the IGT scores across the five blocks from the experiment, with these scores serving as the within-subject factor and group designation (smartphone addiction (SA) group *vs*. healthy control group) as the between-subject factor, a repeated measures ANOVA (rmANOVA) was performed. As depicted in **Figure 4**, the IGT scores for both the SA group and the healthy control group exhibit an ascending trend throughout the five blocks of the IGT task. This upward trajectory suggests an enhancement in decision-making abilities over time.

The healthy group exhibited higher scores in the five blocks compared to the addiction group. The multivariate test results indicated a significant main effect of Block (*F* = 9.972, *p* <.05, *ηp2* = .347), demonstrating significant differences in scores across different task stages, with scores gradually increasing as the Blocks progressed. The interaction effect between Block and Group was not significant (*p* >.05), indicating no interaction between the two factors. The inter-subject effect test showed a significant group effect (*F* = 4.344, *p* <.05, *ηp2* = .053), indicating a significant difference in IGT scores between the addiction group and the healthy group, with the healthy group scoring significantly higher. Pairwise comparisons were conducted to compare the IGT scores among the Blocks.

**Figure 5:**
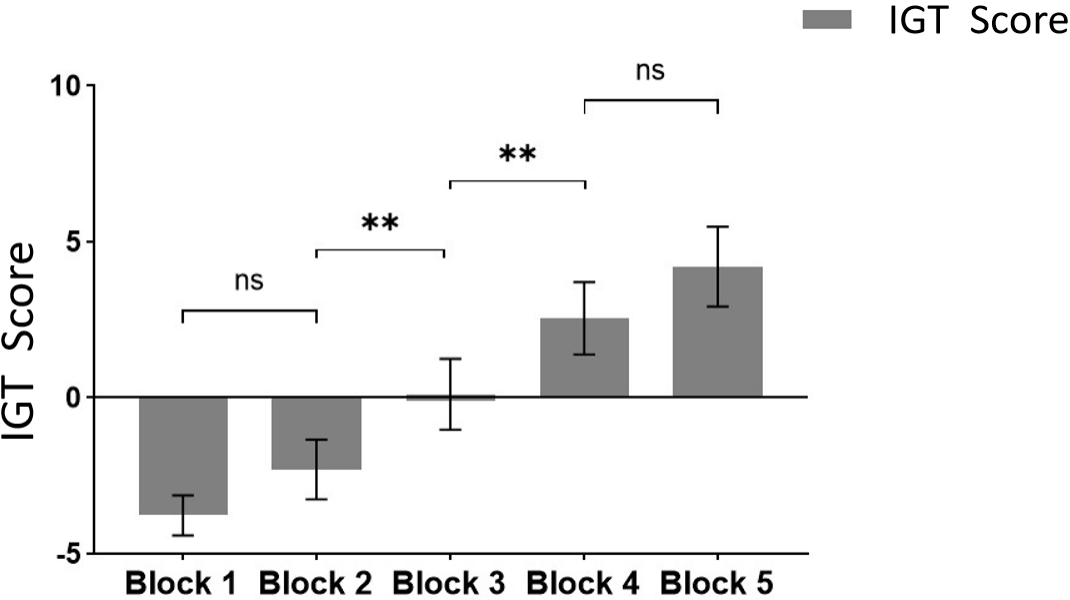
Comparison of IGT scores at different task stages. ** means *p* ≤ 0.001; ns means not significant.

The results from multiple comparisons indicate that there is no significant difference between Block 1 and Block 2 (*p* >.05), whereas a significant difference is observed between Block 2 and Block 3 (*p* <.05), as well as between Block 3 and Block 4 (*p* <.05). However, there is no significant difference between Block 4 and Block 5 (*p* >.05) (see **Figure 5** for reference).

Based on our results, the multivariate test results indicated a significant main effect of Block, with scores gradually increasing as the Blocks progressed. It shows obvious learning effect. In addition, as mentioned above, a significant difference between Block2 and Block3 and a significant difference between Block3 and Block4. Finally, combined with the previous research results (27), Block1 and Block2 (the first 40 Trials) may be designated as the ambiguous selection stage, while Block4 and Block5 (the last 40 Trials) can be categorized as the risk selection stage(To ensure that participants learn the rules of the game and have a clear grasp of decision probabilities, we do not include Block3 in the scope of subsequent analysis) (27).

**Figure 6:**
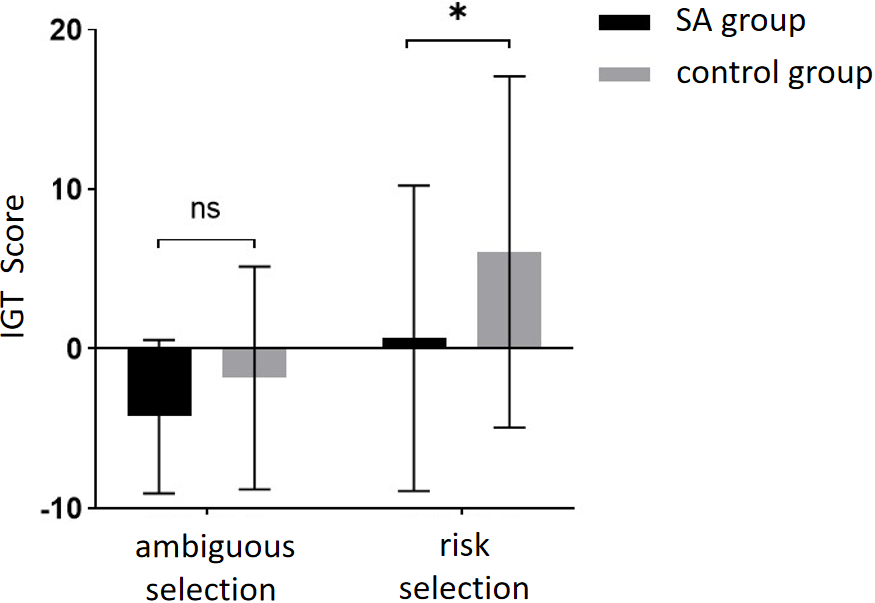
The IGT scores of the SA group and the control group in different decision scenarios. * means *p* ≤ 0.05; ns means not significant.

Finally, an independent student *t*-test was conducted to compare the scores of the two groups at different stages (**Figure 6**). No significant difference was found in IGT scores between the two groups in the ambiguous selection stage (Block 1-2), but a significant difference was observed in the risk selection stage (Block 4-5). The scores of the healthy group were significantly higher than those of the addiction group. These findings suggest that while there was no significant difference between the two groups in the early stages of decision-making, there was a significant difference in the later stages, indicating that individuals with addiction may exhibit impaired decision-making skills when faced with higher levels of risk.

### fNIRS results

**Figure 7:**
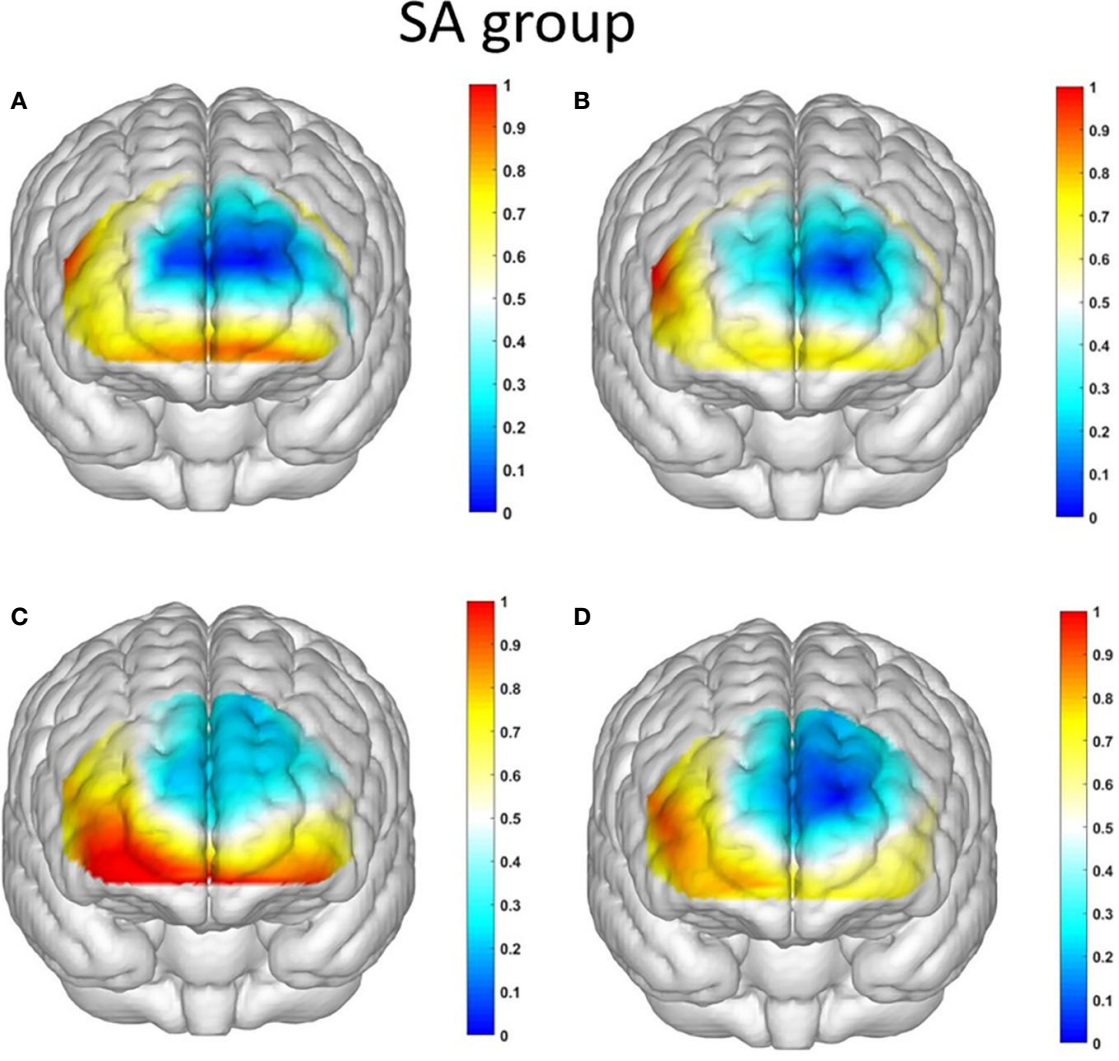
The activation maps of the measured brain regions in the addiction group are presented as follows: **(A)** illustrates the activation map of the addiction group during Condition 1, where an ambiguous situation leads to unfavorable choices; **(B)** displays the activation map of the addiction group during Condition 2, where an ambiguous situation leads to favorable choices; **(C)** demonstrates the activation map of the addiction group during Condition 3, where a risky situation results in unfavorable choices; and **(D)** showcases the activation map of the addiction group during Condition 4, where a risky situation leads to favorable choices.

**Figure 8:**
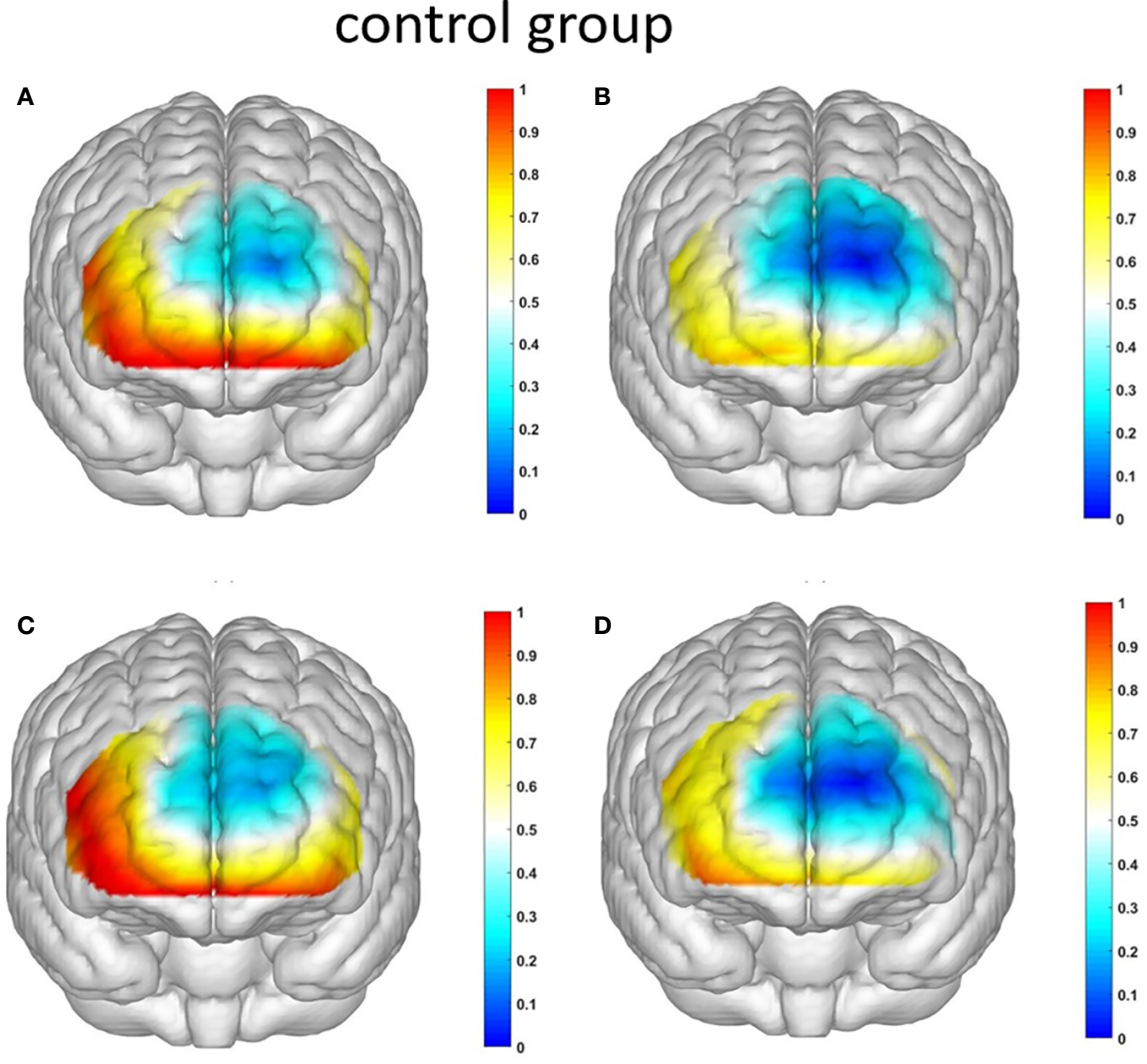
The activation maps of the measured brain regions in the control group are presented as follows: **(A)** illustrates the activation map of the control group during Condition 1, where an ambiguous situation leads to unfavorable choices; **(B)** displays the activation map of the control group during Condition 2, where an ambiguous situation leads to favorable choices; **(C)** demonstrates the activation map of the control group during Condition 3, where a risky situation results in unfavorable choices; and **(D)** showcases the activation map of the control group during Condition 4, where a risky situation leads to favorable choices.

**Figure 9:**
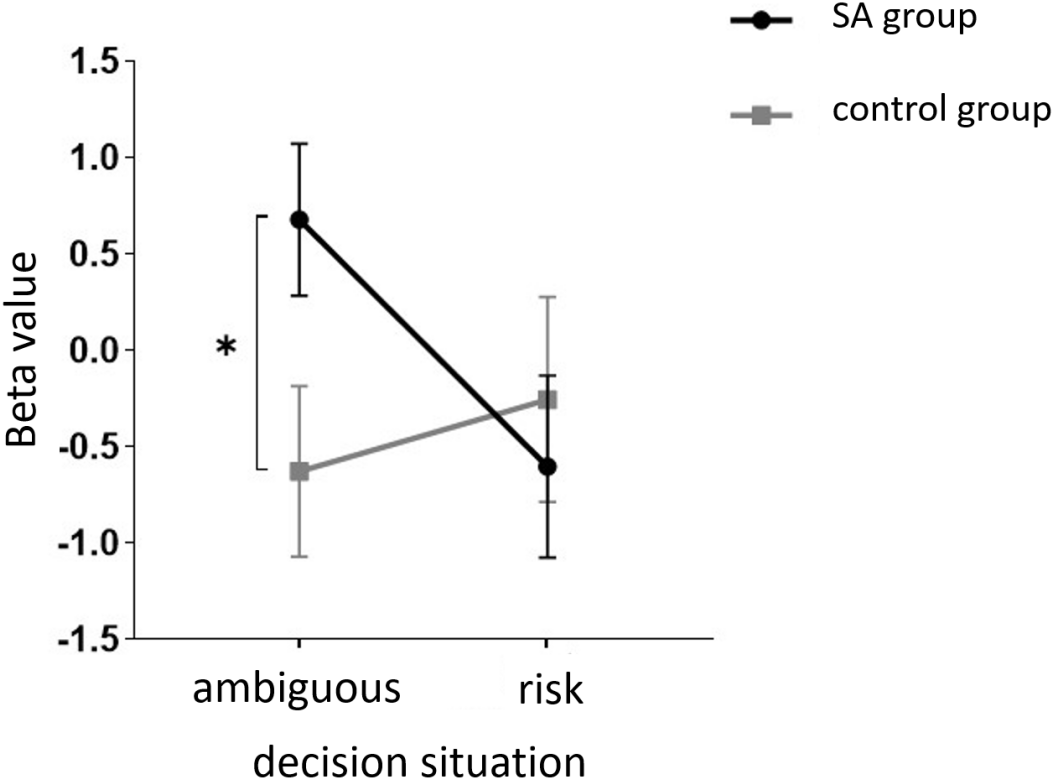
The interaction between two distinct groups in two distinct decision contexts within Channel 4. * means *p* ≤ 0.05.

**Figure 10:**
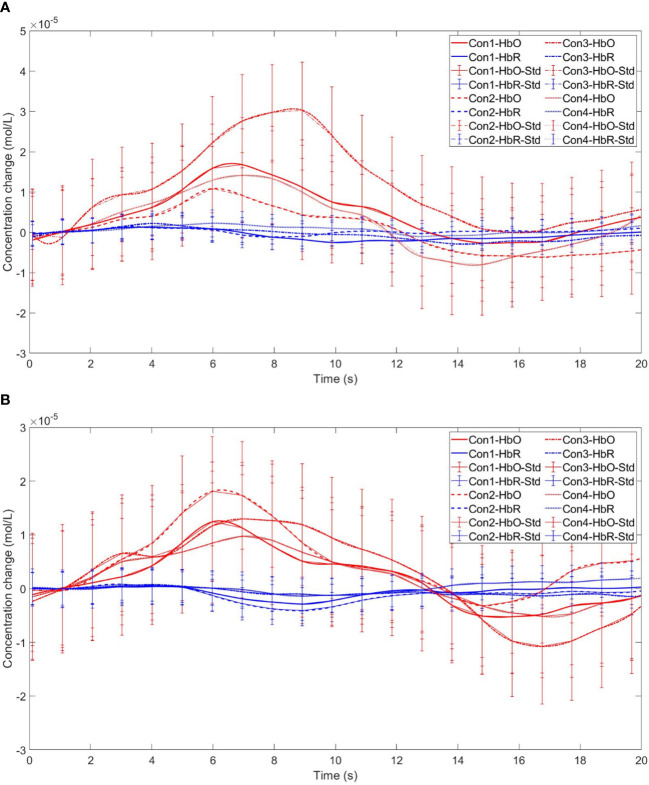
Time series curves of HbO and HbR. **(A)** The time series graphs of HbO and HbR for the addiction group under four conditions in Channel 4; **(B)** The time series graphs of HbO and HbR for the control group under four conditions in Channel 4.

Starting with the examination of channel activity, the results displayed significant activation in certain channels of both the OFC and the dlPFC under the four different task conditions (refer to the **Figures 7** and **8**). In the within-subject effect test, channel 4 revealed a significant interaction between situation and group (*F* = 5.705, *p* <.05, *ηp2* = .077). Simple effect analysis showed that in the addiction group, the ambiguous decision situation was significantly higher than the risk decision situation (*p* < 0.01, *Cohen’s d* = 2.95), and in the control group, there was no significant difference between the two decision situations (*p* = 0.474). Moreover, in the ambiguous decision-making situation, the addiction group was significantly higher than the control group (*p* < 0.05, *Cohen’s d* = 3.12) and in the risk decision scenario, there was no significant difference between the two groups (*p* = 0.626), see **Figures 9** and **10**. Similarly, channel 4 exhibited a significant interaction between choice and group (*F* = 5.705, *p* <.05, *ηp2* = .077), see **Figures 9** and **10**.

**Figure 11:**
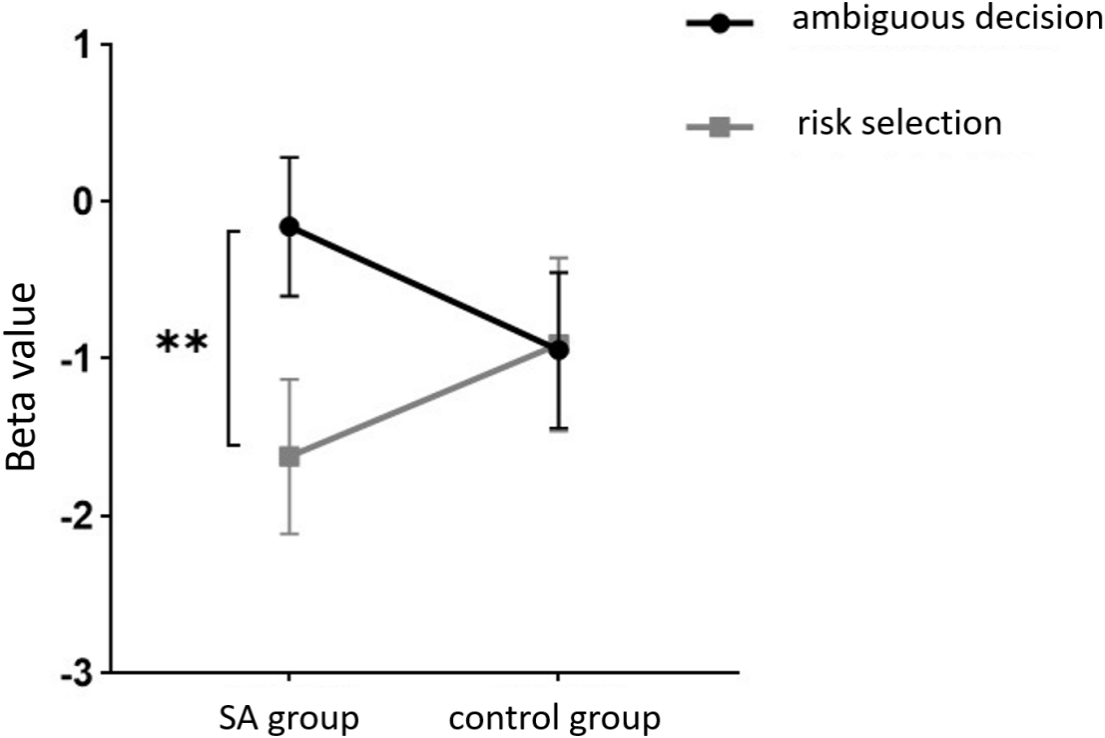
The interaction between two distinct groups in two distinct decision contexts within Channel 9. ** means *p* ≤0.001.

**Figure 12:**
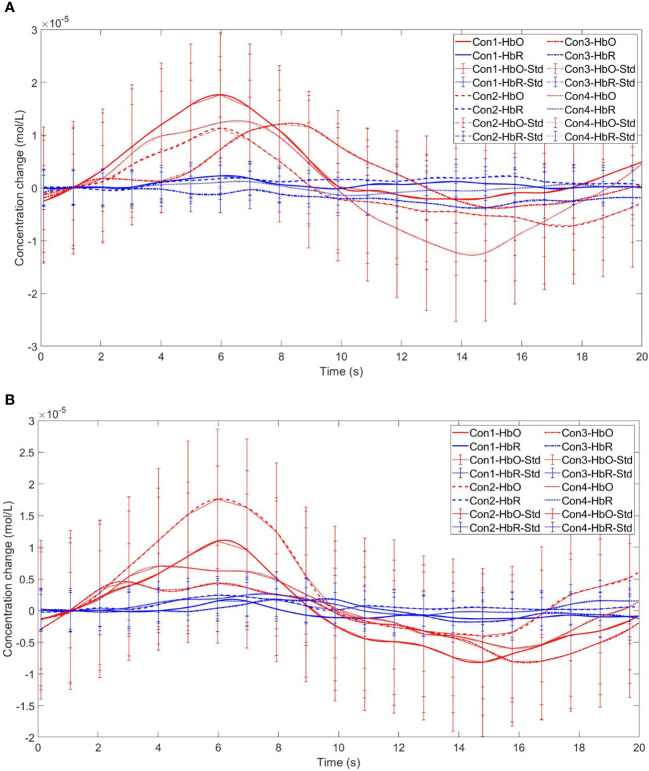
Time series curves of HbO and HbR. **(A)** The time series graphs of HbO and HbR for the addiction group under four conditions in Channel 9; **(B)** The time series graphs of HbO and HbR for the control group under four conditions in Channel 9.

The within-subject effect test for channel 9 demonstrated a significant interaction between situation and group (*F* = 4.431, *p* <.039, *ηp2* = .061). Simple effect analysis showed that in the addiction group, the ambiguous decision situation was significantly higher than the risk decision situation (*p* < 0.01, *Cohen’s d* = 3.13), and in the control group, there was no significant difference between the two decision situations (*p* = 0.944), see **Figures 11** and **12**.

**Figure 13:**
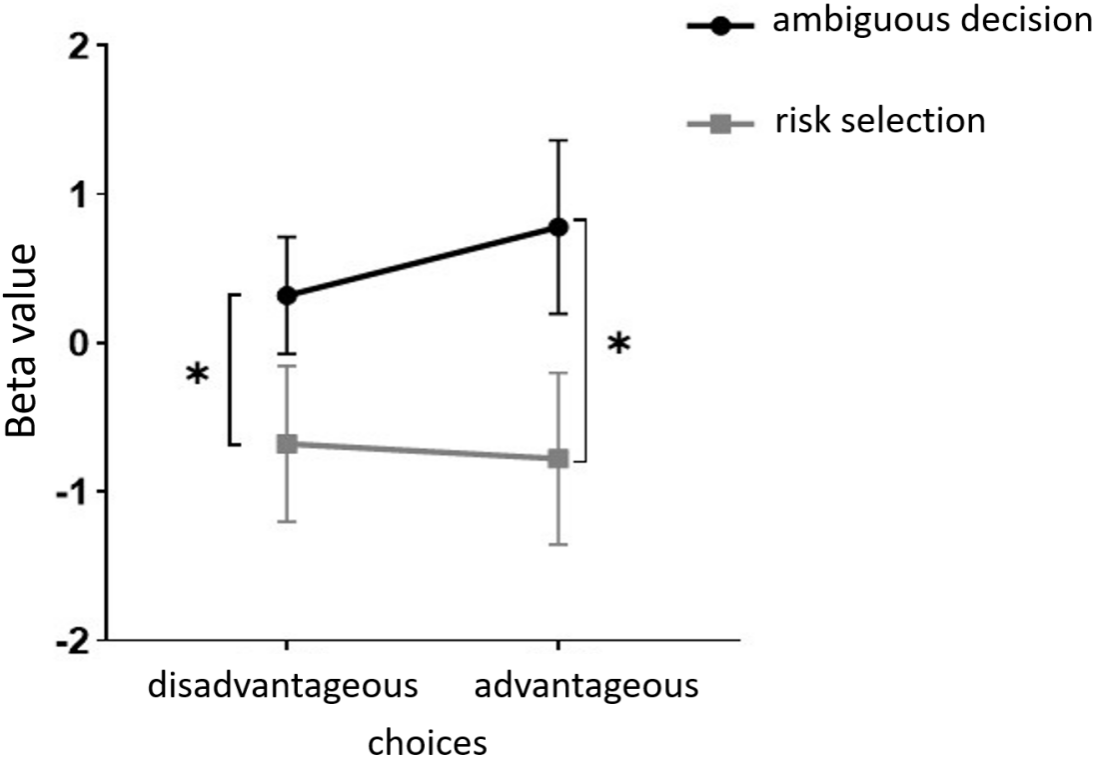
In Channel 11, the interaction between two decision contexts and two choices in the addiction group. * means *p* ≤ 0.05.

**Figure 14:**
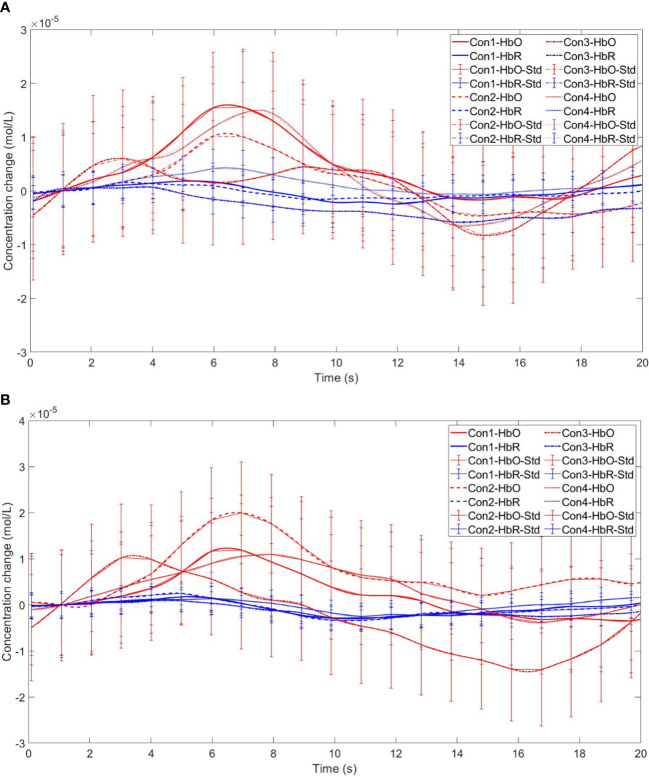
Time series curves of HbO and HbR. **(A)** The time series graphs of HbO and HbR for the addiction group under four conditions in Channel 11; **(B)** The time series graphs of HbO and HbR for the control group under four conditions in Channel 11.

Furthermore, in channel 11, the within-subject effect test indicated a significant interaction between situation and group (*F* = 6.961, *p* <.05, *ηp2* = .093). Simple effect analysis showed that in the addiction group, the ambiguous decision situation was significantly higher than the risk decision situation (*p* < 0.01, *Cohen’s d* = 3.04). In the control group, there was no significant difference between the two decision scenarios (*p* = 0.558). Additionally, there was a significant interaction between situation, choice, and group (*F* = 5.067, *p* <.05, *ηp2* = .069). Further simple effect analysis found that, for the addiction group, the decision situation would affect the individual’s choice. For both unfavorable choice and favorable choice, the individuals in the ambiguous decision situation were higher than those in the risk decision situation (unfavorable choice, *p* < 0.05, *Cohen’s d* = 2.15; favorable choice, *p* < 0.05, *Cohen’s d* = 2.68). For the control group, the decision situation did not affect the difference between unfavorable and favorable choices (unfavorable choice, *p* = 0.149; favorable choice, *p* = 0.065), see **Figures 13** and **14**.

**Figure 15:**
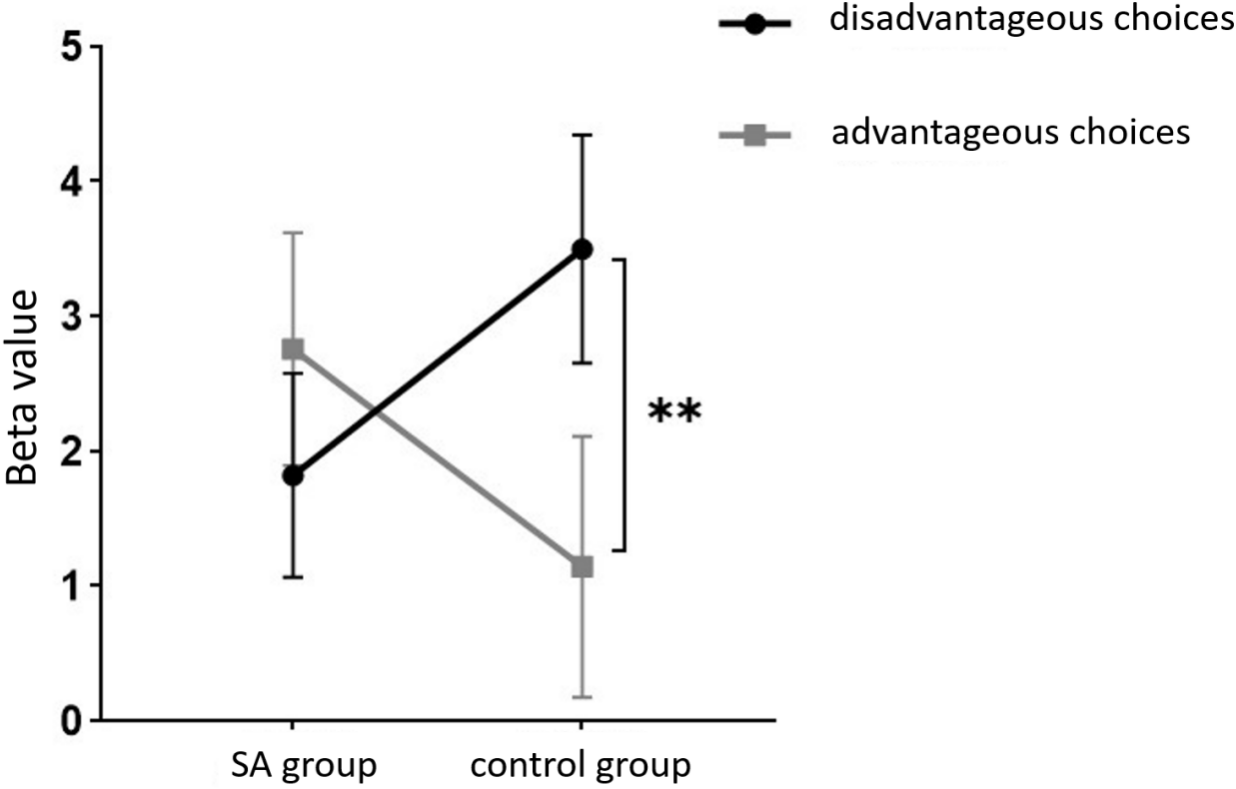
The interaction between two choices and two groups in Channel 18. ** means *p* ≤ 0.001.

**Figure 16:**
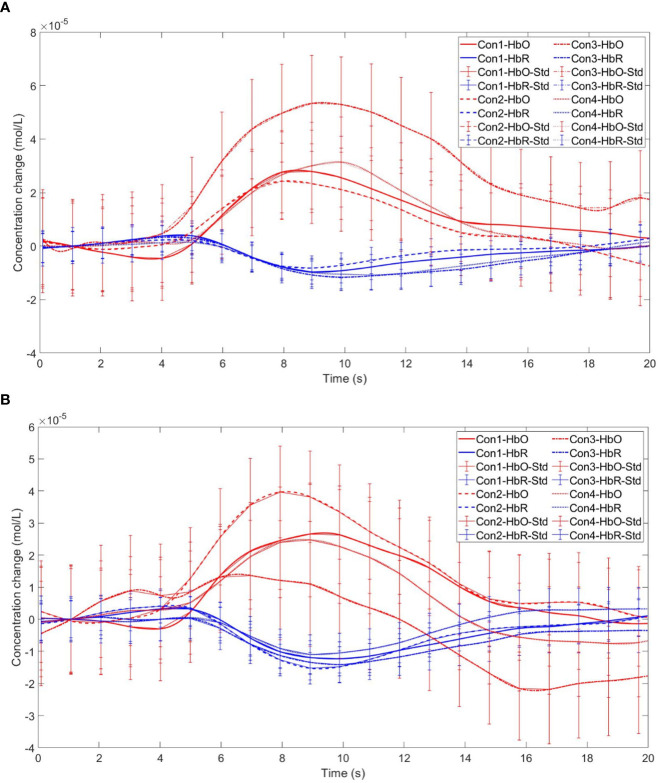
Time series curves of HbO and HbR. **(A)** The time series graphs of HbO and HbR for the addiction group under four conditions in Channel 18; **(B)** The time series graphs of HbO and HbR for the control group under four conditions in Channel 18.

Finally, the within-subject effect test for channel 18 revealed a significant interaction between choice and group (*F* = 8.120, *p* <.01, *ηp2* = .107). Simple effect analysis showed that in the control group, in the control group, individuals had significantly higher Beta values for unfavorable choices compared to favorable choices (*p* < 0.01, *Cohen’s d* = 3.09). In the addicted group, there was no significant difference between the two choices (*p* = 0.228), see **Figures 15** and **16**.

## Discussion

Our investigation into the behavior and brain activity of smartphone addicts during the Iowa Gambling Task (IGT) has revealed three principal findings. First, relative to controls, individuals with smartphone addiction showed elevated levels of smartphone addiction (SA), impulsivity, and depression. Second, while decision-making performance of smartphone addicts was comparable to controls during stages of ambiguity, it was markedly poorer during stages entailing higher risk. Third, brain activation patterns differed between smartphone addicts and the control group, underscoring distinct neural processing in the former.

Our results align with prior research (15, 48, 49), corroborating the association between smartphone addiction and increased depression and impulsivity among college students. However, our findings diverge from studies by (50, 51), regarding anxiety; we observed no significant difference in anxiety levels between smartphone-addicted and non-addicted students. It is important to consider the context of data collection, which occurred during a period of high academic stress due to final exams, potentially elevating and equalizing anxiety across both groups. This may explain the absence of significant disparities in anxiety between the two cohorts.

Our study confirms that college students with smartphone addiction (SA) engage in riskier decision-making behaviors than their healthy counterparts, supporting our hypothesis and aligning with previous findings indicating impaired decision-making in the SA population (14, 52). These students exhibit compromised impulse control, favoring immediate gains over long-term benefits, a hallmark of their decision-making style.

We observed differences in decision-making abilities between the groups at both the ambiguous (early IGT) and risky (late IGT) stages. Notably, no significant differences emerged in the ambiguous stage, yet in the risky stage, the decision-making of smartphone-addicted students was significantly weaker. This contrasts with earlier studies that found impaired decision-making under ambiguity in the SA group (14), but aligns with research suggesting deficits in risky decision-making (32).

Our results suggest that in the ambiguous stage, where outcomes and probabilities remain uncertain, there was no discernible difference in performance between the two groups. This similarity in performance could stem from both groups being in a learning phase and making exploratory choices, thereby exhibiting comparable decision-making capabilities. It is, however, important to emphasize that with increased familiarity and understanding of the consequences of their choices through repeated trials, a shift towards more rational and deliberate decision-making is expected.

In contrast, during the risk decision-making stage, the smartphone addiction group’s Iowa Gambling Task (IGT) scores were significantly lower than those of the control group, indicating a tendency towards more risky and disadvantageous decision-making behaviors. At this juncture, students with smartphone addiction (SA) persisted in their myopic decision-making approach, favoring immediate rewards despite being aware of the adverse long-term outcomes. This inclination towards short-term gratification, particularly in situations fraught with risk, highlights a unique cognitive bias within this group.

From the analysis presented, it is evident that the IGT performance differences between the two groups are intricately linked to their respective decision-making processes.

This research raises concerns as it indicates that college students with smartphone addiction (SA) are consciously engaging in detrimental decision-making without fully considering future repercussions. This behavior aligns with the concept of “short-sightedness” in addiction, where immediate gratification is prioritized, often at the expense of real-world issues and long-term outcomes (14, 53). Individuals with SA may seek instant online feedback, neglecting real-life challenges by immersing themselves in smartphone use (54, 55).

Studies have demonstrated that those with SA show diminished decision-making skills in risky scenarios, potentially due to an overemphasis on rewards and an underestimation of risks. This impaired judgment highlights how SA may disrupt cognitive processes critical for balancing risks and rewards. In the dorsolateral prefrontal cortex (dlPFC), SA students exhibited stronger brain activation in ambiguous decision-making contexts (particularly in channels 4, 9, and 11) compared to risk-based decisions. Specifically, on the left dlPFC (channel 4), SA students had heightened activation during ambiguity but not during risk-related decisions. On the right dlPFC (channel 11), SA students showed increased activation irrespective of outcomes in ambiguous situations compared to risky ones. Moreover, SA students’ brain activation did not significantly vary with the nature of the outcome, unlike healthy individuals who showed more sensitivity towards adverse outcomes (channel 18). These findings reveal that students with SA group exhibit increased activation in the dlPFC during ambiguous decision-making scenarios, but not in situations involving risk. Conversely, for the control group, brain activation did not significantly differ between the two types of decision scenarios. Specifically, we observed that in the left dorsolateral prefrontal cortex (e.g., channel 4), brain activation among college students with smartphone addiction was more pronounced during ambiguous decision-making compared to the control group. We hypothesize that while there are no significant behavioral differences between the two groups during ambiguous decision-making, the smartphone addiction group may display impulsive tendencies (56). Consequently, they might exert more effort in identifying and learning patterns to match the performance level of the control group, leading to higher observed brain activity in the SA group. These results suggest that smartphone addiction influences decision-making in a manner not seen in non-addicted peers. Presumably, individuals without addiction, possibly due to the absence of impulsive traits (57), remain rational and instinctively more cautious about making adverse choices, unlike their addicted counterparts. Thus, no significant difference in brain activation was observed between the two decision-making scenarios among non-addicts. In summary, the disparities in brain activation between the two groups are intricately linked to the decision-making process, highlighting how smartphone addiction uniquely impacts cognitive functions.

Research underscores the critical role of the dorsolateral prefrontal cortex (dlPFC) in the decision-making processes of individuals with smartphone addiction. This area, integral for executive functions such as impulse control, cognitive flexibility, and outcome evaluation, may be compromised by smartphone addiction, potentially leading to impaired decision-making. Notably, those with smartphone addiction often show increased dlPFC activation during ambiguous decision-making scenarios, regardless of the potential benefits or detriments of the choices presented. This indicates a possible difficulty in effectively processing and appraising uncertain situations.

The relationship between smartphone addiction and decision-making in risky contexts, however, is not yet clear. Some evidence points to a heightened sensitivity to potential losses in individuals with smartphone addiction, marked by increased dlPFC activation, which could lead to a more risk-averse and cautious approach. Yet, further studies are essential to clarify the patterns and neural underpinnings of decision-making in risky contexts for those with smartphone addiction.

This study’s insights are particularly relevant for college students, a group for whom decision-making carries substantial long-term consequences. The findings serve as a caution, demonstrating the adverse impact of smartphone addiction on decision-making and underscoring the need for strategies to address this issue. Educational institutions could incorporate psychological interventions to reduce the risks associated with excessive smartphone use.

Recognizing the limitations of this study is essential. Due to its cross-sectional design, it is not possible to draw causal conclusions. Additionally, the timing of data collection, which occurred during the final year of college, may have introduced specific situational factors that could affect the results. Furthermore, our analysis concentrated on changes in HbO without examining HbR, potentially overlooking significant insights. Lastly, although the fNIRS data provide valuable information on patterns of brain activation, they constitute only a single component of a more complex picture. Further research is necessary to enhance our comprehension of these phenomena comprehensively.

## Conclusion

In conclusion, the decision-making capabilities of individuals grappling with smartphone addiction appear to be context-dependent, exhibiting distinct patterns based on the nature of the decision-making scenario. The influence of smartphone addiction on behavior, especially within the dorsolateral prefrontal cortex (dlPFC)—a region pivotal for executive functions—is particularly significant. This region’s altered activation in those with smartphone addiction underscores the potential disruption of cognitive processes essential for sound decision-making.

While there is a clear indication that the dlPFC’s functioning is affected during ambiguous decision-making situations, the specific behavioral and neural mechanisms at play when individuals with smartphone addiction face risky decisions remain elusive. This gap in our understanding points to the need for a more nuanced examination of how such individuals weigh potential gains against losses, and how their risk assessment might differ from non-addicted peers.

Future research endeavors should prioritize a comprehensive investigation into the multifaceted impact of smartphone addiction on decision-making. This includes a deeper dive into risk-related decisions, which are particularly relevant given the real-world implications of poor risk management. By expanding our knowledge in this domain, we can better understand the cognitive and neural dynamics of smartphone addiction, which is crucial for developing targeted interventions to mitigate its adverse effects. Furthermore, such research could inform the creation of educational programs and therapeutic strategies designed to enhance decision-making skills and promote healthier behavioral patterns among those affected by smartphone addiction.

## Data availability statement

The raw data supporting the conclusions of this article will be made available by the authors, without undue reservation.

## Ethics statement

The studies involving humans were approved by Institutional Review Board of Sichuan Normal University. The studies were conducted in accordance with the local legislation and institutional requirements. The participants provided their written informed consent to participate in this study. Written informed consent was obtained from the individual(s) for the publication of any potentially identifiable images or data included in this article.

## Author contributions

XL: Funding acquisition, Methodology, Project administration, Supervision, Validation, Writing – review & editing. RT: Data curation, Formal Analysis, Investigation, Validation, Visualization, Writing – original draft. XB: Data curation, Formal Analysis, Writing – review & editing. HL: Conceptualization, Formal Analysis, Investigation, Writing – review & editing, Writing – original draft. TL: Validation, Visualization, Writing – review & editing. XZ: Methodology, Supervision, Validation, Writing – review & editing. YL: Funding acquisition, Project administration, Writing – review & editing.
